# Arsenic Exposure During Pregnancy and Childhood: Factors Explaining Changes over a Decade

**DOI:** 10.3390/toxics13030215

**Published:** 2025-03-16

**Authors:** Paola Rubilar, Macarena Hirmas-Adauy, Mauricio Apablaza, Camila Awad, Xaviera Molina, María Pía Muñoz, Iris Delgado, Nicolás C. Zanetta-Colombo, Carla Castillo-Laborde, María Isabel Matute, Mauricio A. Retamal, Andrea Olea, Paulina Pino, Claudia González, Cristóbal Carvajal, Verónica Iglesias

**Affiliations:** 1 Centro de Epidemiología y Políticas de Salud, Facultad de Medicina Clínica Alemana, Universidad del Desarrollo, Santiago 7610658, Chile; mhirmas@udd.cl (M.H.-A.); c.awad@udd.cl (C.A.); xmolinaa@udd.cl (X.M.); idelgado@udd.cl (I.D.); carlacastillo@udd.cl (C.C.-L.); mimatute@udd.cl (M.I.M.); aolea@udd.cl (A.O.); claudiagonzalez@udd.cl (C.G.); 2 Facultad de Gobierno, Universidad del Desarrollo, Santiago 7610658, Chile; mapablaza@udd.cl; 3 Programa de Epidemiología, Escuela de Salud Pública, Facultad de Medicina, Universidad de Chile, Santiago 8380453, Chile; maria.munoz.qu@uchile.cl (M.P.M.); ppino@uchile.cl (P.P.); 4 Programa de Doctorado en Salud Pública, Escuela de Salud Pública, Facultad de Medicina, Universidad de Chile, Santiago 8380453, Chile; 5 Department of Geography, South Asia Institute, Heidelberg University, 69120 Heidelberg, Germany; nicolas.zanetta@stud.uni-heidelberg.de; 6 Heidelberg Center for the Environment (HCE), Heidelberg University, 69120 Heidelberg, Germany; 7 Programa de Comunicación Celular en Cáncer, Instituto de Ciencias e Innovación en Medicina, Facultad de Medicina Clínica Alemana, Universidad del Desarrollo, Santiago 7610658, Chile; mretamal@udd.cl; 8 Centro de Informática Biomédica, Instituto de Ciencias e Innovación en Medicina, Facultad de Medicina Clínica Alemana, Universidad del Desarrollo, Santiago 7610658, Chile; cristobalcar@gmail.com

**Keywords:** inorganic arsenic, prenatal exposure, biomarker, exposure in children, environmental justice, water exposure

## Abstract

Arsenic chronic exposure, particularly in its inorganic form, represents a significant public health concern. This study was conducted in Arica, the northernmost city in the country, whose inhabitants have been exposed to inorganic arsenic both naturally through drinking water and anthropogenically due to a toxic waste disposal site. We explored changes in inorganic arsenic levels in a cohort of pregnant women and their children over a decade, identifying exposure trends and their determinants. We used data on arsenic exposure through maternal urine samples during pregnancy, collected by the Health Authority between 2013 and 2016 (measurement 1), and followed up with assessments of their children in 2023 (measurement 2). Temporal changes in inorganic arsenic concentration were analyzed using the Wilcoxon Signed-Rank test, and a mixed linear regression model was employed to determine which factors contributed to urinary inorganic arsenic levels. We did not observe significant differences in mean arsenic concentrations between the two-time points (*p* = 0.4026). The mixed linear regression model revealed that children consuming bottled water had 8.3% lower urinary inorganic arsenic concentrations than those drinking tap water (95% CI: −15.36 to −0.54%). Additionally, children from ethnic groups had 8.64% higher inorganic arsenic concentrations (95% CI: 0.49 to 17.5%), while those with caregivers with higher education showed a 13.67% reduction (95% CI: −25.06 to −0.56%). Despite mitigation efforts, these findings underscore the ongoing risk of inorganic arsenic exposure among vulnerable populations. They further emphasize the importance of addressing natural arsenic contamination in water and implementing targeted interventions to reduce disparities associated with socioeconomic and demographic factors.

## 1. Introduction

Arsenic exposure is a global public health problem. Arsenic, in its inorganic form, is naturally found in the groundwater of many countries and is highly toxic. The main threat comes from using water contaminated with arsenic for human consumption, cooking, and the irrigation of vegetables [1]. Exposure can also result from anthropogenic pollution, mainly from mining and metallurgic activities or industrial waste deposition [2]. Arsenic can cross the placental barrier during pregnancy when the fetus is still developing, which can be risky for fetal and child health, in addition to generating premature diseases in adulthood [1,3,4,5]. This relates to the concept of Developmental Origins of Health and Disease (DOHaD), which suggests that certain environmental factors during critical stages of development can influence tissue formation and function, leading to adverse health effects throughout life [6].

In 2001, the World Health Organization (WHO) set a limit of 10 µg/L (0.01 mg/L) of arsenic in safe drinking water to protect public health. However, evidence indicates that arsenic can cause harm even at concentrations below 10 µg/L [7,8], especially among vulnerable populations [2,9,10], who often cannot protect against arsenic contamination. In urban settings, low-income and marginalized communities usually reside in neighborhoods with inadequate water and sewage systems, higher pollution levels, proximity to industrial or mining waste sites, and less access to healthcare and environmental information [11,12]. The lack of policies and investment in health infrastructure perpetuates toxic exposure, increasing health disparities and the burden of arsenic-related diseases [2,13]. There is concern about whether current safety standards are adequate, especially for vulnerable populations [2,9,10]. 

This research was conducted in Arica, the northernmost city of Chile, where 98.2% of the region’s population lives [14]. Arica is in a geological area rich in minerals, where arsenic is naturally present in rock formations and leaches into groundwater [15]. In the 1980s, over 20,000 tons of toxic waste was imported for recycling and abandoned in an urban area of the city [16]. Years later, housing complexes were built near this area. An analysis of the composition of the toxic waste showed high concentrations of heavy metals, including arsenic and lead. This situation led to a public health and environmental crisis, prompting Law No. 20,590 in 2012, which considered interventions and the monitoring of the exposed population [17]. This study explores changes in inorganic arsenic levels among expectant mothers and their children over a decade, identifying exposure trends and their determinants. Specifically, we will evaluate socioeconomic and environmental determinants of inorganic arsenic concentrations.

## 2. Methods

### 2.1. Design and Study Population

This article presents a follow-up study of a pregnant women–child cohort. Baseline data were collected by the Health Authority of the Region of Arica and Parinacota between 2013 and 2016 from 1644 pregnant women receiving care at the Regional Hospital of Arica before delivery (measurement 1). During that period, surveys were administered, and inorganic arsenic concentrations in urine samples were measured. The Health Authority provided this information as part of a collaboration agreement.

The original sample size was calculated, assuming a blood lead prevalence of 1% (samples > 10 µg/dL) and inorganic arsenic prevalence in urine >35 µg/L of 12%, with margins of error of <0.5% for lead and <2% for arsenic, resulting in a sample size of 1519 mother-newborn pairs, accounting for a 10% loss rate.

Measurement 2 was carried out in 2023 as part of a study titled “Arsenic exposure and its association with proinflammatory cytokines in children born between 2013 and 2016 in Arica”. For this purpose, 980 women (59.6% of the original cohort) were successfully re-contacted, and 782 agreed to participate and met the inclusion criterion of still residing in the city, representing 47.6% of the original sample and 79.8% of those re-contacted. A random subsample of mothers was selected, resulting in a cohort of 450 children aged 7 to 10 (seven mothers had multiple births).

Comparison between the entire sample of measurement 1 (1644 pregnant women) and the subsample of mothers enrolled in measurement 2 (443) showed similar maternal characteristics and no differences in the distribution of inorganic arsenic concentration (see Supplementary Materials). In 2023, both mothers and children provided informed consent and assent, respectively. Additionally, a survey was administered to the adult responsible for the child at enrollment.

### 2.2. Inorganic Arsenic Exposure

In the first measurement (2013–2016), trained healthcare personnel collected a spot urine sample from each mother during prenatal care visits or hospitalization before delivery. Inorganic arsenic and its methylated metabolites were analyzed using atomic absorption spectrophotometry with hydride generation (AAS-HG) at the Occupational Health Laboratory of the Institute of Public Health laboratory (ISO/IEC 17025:2017) [18]. The limit of detection (LOD) was 5 µg/L, and concentrations below the LOD (10.6%) were assigned a value of 2.5 µg/L (LOD/2). Information on the urinary concentration of inorganic arsenic and its methylated metabolites in pregnant women as well as the urinary creatinine concentration was provided by the Arica and Parinacota Region Health Authority.

For the second measurement (2023), a trained nursing assistant visited each child’s residence to collect a 50 mL spot urine sample. This visit was scheduled one or two days in advance, during which participants received specific instructions which were reinforced on the day of the visit. Caregivers were instructed to ensure children avoided liquids or urination for four hours before the appointment and abstained from seafood consumption for 72 h. The urine samples were code-labeled, stored at −4 °C, and delivered to an accredited private laboratory [19]. The analyses were performed within the same week using inductively coupled plasma mass spectrometry (Perkin Elmer NexION^®^ 2000 ICP-MS), allowing arsenic speciation and the individual determination of each species of interest: arsenite (AsIII), arsenate (AsV), monomethylarsonic acid (MMA), dimethylarsinic acid (DMA), and arsenobetaine. The detection limit for each analyte was 0.1 µg/L. In this second measurement, we summed the inorganic arsenic species (AsIII and AsV) and methylated arsenic species (MMA and DMA) as a measurement of “inorganic arsenic concentration” based on evidence establishing it as a reliable biomarker for assessing recent inorganic arsenic exposure from multiple sources [20,21,22,23]. All samples had inorganic arsenic values above the detection limit. Urinary creatinine concentration was also determined in the same laboratory.

### 2.3. Covariates

Sociodemographic information like age, ethnic group, and years of education were collected through a survey, along with health history data such as comorbidities, body mass index, and maternal tobacco use. Additionally, risk factors related to arsenic exposure were assessed, including living on paved streets, using pesticides or derivatives within the household, and consuming fish three days before urine sampling. During the first measurement, participants were asked if they lived in an area classified as at risk by the Health Authority. In the second measurement, they were asked whether they were beneficiaries of Law 20,590, which primarily applies to those who had lived in high-risk areas [24]. Additionally, the second measurement gathered information on the drinking water source used by both the child and the mother during pregnancy.

### 2.4. Statistical Analysis

Unadjusted and creatinine-adjusted inorganic arsenic concentration (for values between 0.3 and 3.0 gr/L) were described using descriptive statistics, including maximum and minimum values, 25th, 50th, 75th, 90th, and 95th percentiles, arithmetic mean, standard deviation, and geometric mean. Creatinine adjustment reduced the sample size by 405 mothers and 408 children.

Differences in creatinine-adjusted inorganic arsenic concentration between measurements 1 and 2 were compared with the Wilcoxon Signed-Rank test. We use the same test to compare the creatinine-adjusted inorganic arsenic concentration in specific subgroups of the study, such as pregnant women who lived in the area considered at risk of exposure to toxic waste in measurement 1, and the inorganic arsenic concentration of their children in measurement 2. Similarly, the inorganic arsenic concentration of children whose parents were covered by Law 20.590, established in measurement 2, was compared with the inorganic arsenic concentration of their mothers during pregnancy, established in measurement 1.

Due to the non-normal distribution of inorganic arsenic concentrations, values were transformed to a logarithmic scale. Spearman’s correlation coefficient was used to evaluate the relationship between the concentration of inorganic arsenic in the mother and their children. A bivariate analysis of creatinine-adjusted inorganic arsenic concentration was conducted using the Wilcoxon, Mann–Whitney, and Kruskal–Wallis tests to explore differences across sociodemographic, health, and exposure-related factors.

A log-transformed linear mixed model for inorganic arsenic was implemented to assess the factors explaining the baseline inorganic arsenic exposure and its trajectory over time. Covariates with a *p*-value <0.10 in the bivariate analysis were included in the model: ethnicity, schooling, the age of the child, a change of address since birth, drinking water source, the use of pesticide, and living on a street with paving. Since these last three correspond to risk variables for arsenic exposure, they were assessed using information from measurements 1 and 2. The random effect of the model was given by the individual (child). The intraclass correlation (ICC) was calculated to assess the variance decomposition. The model is as follows:

$$\begin{array}{ll} \log\;inorganic\;arsenic\;(Y_{ij}) & \;=\beta_{0}+\beta_{1}drinking\;water+\beta_{2}\;belongs\;to\;ethnic+\beta_{3}schooling+\beta_{4}use\;of\;pesticides\\ & \;+\beta_{5}paved\;street+\beta_{6}age\;of\;the\;child+\beta_{7}\;change\;of\;address+u_{0j}+\epsilon_{ij} \end{array}$$

To improve the interpretation of the coefficients, the beta values were transformed using the following formula:

*Percentage* = (*exp*(*β*) − 1) × 100

The statistical analyses were conducted using R (version 4.2.3) within R Studio. 

## 3. Results

### 3.1. Characteristics of Pregnant Women in Measurement 1 and Their Children in Measurement 2

Table 1 describes the current study sample, including the characteristics of the pregnant women during measurement 1 (2013–2016) and the characteristics of their children in 2023 (measurement 2). The children’s survey was answered by the adult responsible for the child, which in 86.4% of cases was the mother, in 6.4% of cases the father, and in 7.1% of cases another caregiver (sibling, grandparent, or uncle/aunt).

In measurement 1, 84.2% of the pregnant women were between 20 and 39 years old, with a high prevalence of overweight and obesity (85.2%) before pregnancy. Of these women, 39% reported belonging to an ethnic group, which increased to 45.3% among their children in measurement 2. Regarding educational attainment, only 16.3% of the pregnant women reported completing more than 12 years of education in measure 1, compared to 34% of the caregivers in 2023. Pesticide use increased significantly, from 2.7% of mothers during pregnancy to 47.6% in measurement 2. The street pavement remained consistent at around 80%, while 34.7% of the children had changed residences since birth. Significant changes were observed in drinking water sources, with a decrease in tap water use, an increase in bottled water consumption, and a further reduction in the use of well water in measurement 2. Regarding residence in areas at risk of soil contamination, as asked in measurement 1, 9.7% of the mothers lived in such areas. In measurement 2, 16% of the children had at least one parent registered as a beneficiary of the law in question.

### 3.2. Inorganic Arsenic Concentration During Pregnancy and Childhood

The median concentrations of creatinine-corrected inorganic arsenic were 17.0 µg/g for pregnant women (measurement 1) and 16.3 µg/g for children (measurement 2), with geometric means of 16.9 µg/g and 16.6 µg/g, respectively. No significant differences were observed between the two measurements over time (Wilcoxon Signed-Rank test *p*-value = 0.4026). Similarly, no significant differences were found in the median concentration of inorganic arsenic between measurements 1 and 2 among pregnant women who were living in the exposure area (Wilcoxon Signed-Rank test *p*-value = 0.3042) or among children whose parents were beneficiaries of the Law No. 20,590 (Wilcoxon Signed-Rank test *p*-value = 0.8015). However, a lower median concentration of inorganic arsenic measured in 2023 was observed in this group (Table 2).

A positive correlation was observed between the log-transformed inorganic arsenic concentration in pregnant women and children (r = 0.2138; *p* < 0.0001).

### 3.3. Bivariate Analysis Between Study Variables and Inorganic Arsenic Concentration in Pregnant Women and Children

Table 3 shows that pregnant women who self-identified as belonging to an ethnic group had a higher median concentration of inorganic arsenic than those who did not (18.4 vs. 16.3 µg/g). Similarly, children reported as belonging to an ethnic group also exhibited higher concentrations (*p*-value = 0.009). Both in pregnant women during measurement 1 and the adults responsible for the children during measurement 2, lower years of education were associated with a higher median concentration of inorganic arsenic.

Significant differences were observed in the water sources used for consumption by pregnant women (measurement 1) and children (measurement 2). Well water or rural potable water consumption had the highest median arsenic concentrations, while bottled water consumption was associated with lower levels than tap water in both measurement periods. An inverse relationship was observed among children between age and median inorganic arsenic concentration, where older age corresponded to lower arsenic levels (*p*-value = 0.0256). Furthermore, the use of pesticides and the absence of paved streets around the children’s homes were associated with higher median inorganic arsenic concentrations.

### 3.4. Multivariate Model

Table 4 presents the factors explaining variations in inorganic arsenic concentration during pregnancy (measurement 1) and approximately 10 years later (measurement 2). Children who consumed bottled water during measurement 2 exhibited an 8.25% decrease in inorganic arsenic concentration compared to those who drank tap water, a statistically significant result. In contrast, children who consumed well water, rural potable water (APR), or other informal water sources showed a 35.18% higher inorganic arsenic concentration than those who drank tap water; however, this finding was not statistically significant. Individuals who identified as belonging to an ethnic group had an 8.64% higher inorganic arsenic concentration compared to those who did not identify with any group. In addition, children whose responsible adults had more years of education showed a reduction in the inorganic arsenic concentration of 13.67%.

Factors such as the use of pesticides or derivatives in the household, the street pavement where the household resides, a change of address, and the child’s age did not explain the variations in inorganic arsenic concentration in the two measurements.

The intraclass correlation coefficient (ICC) value was 0.19, meaning that 19% of the total variability in inorganic arsenic concentrations could be attributed to inter-individual variability. The remaining 81% was attributable to intra-individual variability.

## 4. Discussion

The findings reveal that inorganic arsenic concentration in pregnant women and their children, measured approximately 10 years apart, does not change significantly. This persistence is consistent with previous longitudinal studies [23] and highlights ongoing arsenic exposure due to natural geological processes [25]. The latest National Health Survey reported a mean urinary arsenic concentration of 16.3 µg/L in the northern macrozone, where Arica is located. This value is similar to the findings of this study and significantly higher than those reported in the metropolitan, central, and southern macrozones (6.7; 8.8; and 9.2 µg/L, respectively) [26]. This reflects natural conditions in some areas. Within the framework of Law 20,590, interventions have been implemented, such as the provision of subsidies for housing improvements, the relocation of families living in high-risk areas, the paving of streets, and implementation of a sanitary control program for affected populations, among others [24]. Additionally, there have been improvements in the quality of potable water and the increased oversight of water treatment plants [27]. Despite the above, no significant changes were observed in the mean inorganic arsenic concentration in the study sample between the two periods. Similarly, no differences were found in inorganic arsenic concentrations between individuals who reported living in areas at risk of exposure at measurement 1 and those who reported being beneficiaries of the law at measurement 2. Studies have reported favorable outcomes following the adoption of regulations to reduce the arsenic maximum contaminant level in drinking water from 50 to 10 µg/L [28], and Chile has been no exception [29]. However, the continued exposure to low doses of arsenic, coupled with populations more susceptible to arsenic-related health effects, highlights the need for more effective and sustainable approaches to mitigate this public health threat [30].

The main determinants of change in inorganic arsenic concentration over the study period include sources of drinking water, ethnicity, and education level. Bottled water usage increased, serving as the primary drinking water source for approximately 80% of the sample due to public health recommendations and increased awareness of exposure risks. Individuals consuming bottled water had an 8.25% reduction in inorganic arsenic concentration compared to those using tap water, indicating a protective effect. This finding can be explained by Chilean regulations, which ensure that bottled water meets the same standards as public water supplies [31]. However, a study suggested that 30% of bottled water samples in Chile exceed the recommended arsenic concentration [32]. Conversely, rural potable water, wells, and other informal supplies were associated with higher inorganic arsenic concentrations than tap water. These sources were grouped together statistically due to the low number of users, revealing a pattern of higher arsenic concentrations. It is important to note that these water sources adhere to different quality standards. In Chile, rural potable water is regulated and monitored under a specific legal framework [33] and must comply with water quality standards [34]. However, rural water quality is not always adequate, with challenges in regulating specific components [35]. On the other hand, well water forms part of the informal water supply in semi-concentrated or dispersed rural populations, and is often associated with poverty [36]. Rural potable water systems in Arica often maintain arsenic concentrations near or above the recommended limits in some treatment plants. This situation is particularly relevant as studies have indicated that even arsenic concentrations close to the recommended limit pose a health risk [37,38]. The World Health Organization (WHO) recommends keeping arsenic concentrations in water as low as possible and below the provisional guideline wherever resources allow [39]. Based on this recommendation, some countries, like the United States, Denmark, and The Netherlands, have demonstrated that stricter arsenic limits are achievable with a robust regulatory framework, investment in water treatment technologies, and a commitment to public health protection [40].

This study shows how arsenic exposure is deeply linked with broader patterns of environmental injustice [41]. Pregnant women or caregivers, especially those from ethnic groups or with lower education attainment, exhibited higher inorganic arsenic concentrations. These findings align with global trends, where marginalized populations disproportionately face environmental hazards due to inadequate regulatory oversight [42]. The persistence of arsenic exposure despite interventions underscores the need to view these issues within the framework of structural inequalities rather than as isolated environmental problems. Indigenous communities and rural populations, often excluded from political representation and resources, remain particularly vulnerable to environmental risks [43]. Addressing these challenges requires the recognition of how intersecting factors—such as gender, ethnicity, and socioeconomic status—determine exposure risks and health outcomes. Achieving environmental justice demands more than technical solutions like water quality regulations. It requires tackling systemic inequalities through environmental monitoring, comprehensive education, and community-led strategies. Only by addressing these primary causes can we create safe environments for marginalized groups and work toward a more equitable and sustainable future [44].

The main strength of this study is its efforts to measure inorganic arsenic concentrations in a subsample of children, where original data were obtained from their mothers during pregnancy. This enables the tracking of inorganic arsenic exposure trajectories over approximately 10 years, providing critical information regarding changes in exposure levels in a population affected by natural and anthropogenic sources. It also helps to identify key determinants and assess the effects of interventions and changes in contamination sources. Approximately 58.5% of the mothers were successfully identified after 10 years, of whom 46.7% agreed to participate, a proportion consistent with another cohort study in the middle of this timeframe [45]. Additionally, the mean concentration of inorganic arsenic among women who participated in the second measurement was similar to that of the original sample, reducing the risk of selection bias.

One limitation of this study is the use of different analytical techniques to measure inorganic arsenic at two different time points, reflecting the technology available during each period. The inductively coupled plasma mass spectrometry (ICP-MS) technique offers greater sensitivity and can detect low concentrations of arsenic. In this study, no samples fell below the detection limit when analyzed with ICP-MS, whereas, with the atomic absorption spectrophotometry (AAS-HG) technique, 10.6% of samples were below the detection limit. Nevertheless, both methods are accurate and robust [46]. A second limitation could be associated with recall bias in the question regarding water consumption during pregnancy, which was included in the most recent survey. However, given that the mothers are highly aware of the city’s contamination issues, this bias will likely be less significant. It is important to highlight that the homes of the pregnant women who participated in the first measurement (*n* = 1644) were evenly distributed throughout the city. However, as part of the implemented interventions, most families living in exposed areas, due to the proximity of their homes to former toxic waste deposits, were relocated. Other families later informally occupied these homes, most of them immigrants. Consequently, the study conducted in 2023 may not accurately reflect the exposure of children currently residing in areas considered to be more exposed.

The assessment of arsenic exposure requires us to consider not only socioeconomic and environmental determinants but also genetic factors, particularly AS3MT gene expression, which plays a protective role in arsenic metabolism and excretion [47]. Incorporating genetic factors into future research could be helpful in determining groups more susceptible to the toxic effects of arsenic, especially in regions with high historic arsenic exposure, such as northern Chile.

## 5. Conclusions

In conclusion, this study shows that exposure to inorganic arsenic in Arica has remained sustained over the last decade, highlighting the socioeconomic and environmental inequalities that influence this exposure. The evidence of the effects of low-dose exposure on health emphasize the importance of addressing natural arsenic contamination in water through stricter regulations and targeted interventions to reduce disparities associated with socioeconomic and demographic factors.

## Figures and Tables

**Table 1:** Sociodemographic and risk characteristics of arsenic exposure in pregnant women measured in 2013–2016 and their children in 2023, Arica, (Chile).

	Sample Size (Pregnant Women/Children)		Measurement 1 (Pregnant Women)		Measurement 2 (Children)	
			***n***	**%**	***n***	**%**
Age of the mother	443	Under 20 years of age	61	13.8		
		20 to 39 years old	373	84.2		
		40 years old or older	9	2		
Age of the children	450	7 years old			146	32.4
		8 years old			233	51.8
		9 years old or older			71	15.8
Sex		Female			223	49.6
		Male			227	50.4
Body mass index	441/448	Malnourished	0	0	21	4.7
		Normal weight	65	14.7	178	39.7
		Overweight	167	37.8	101	22.5
		Obese	209	47.4	148	33.0
Belongs to ethnic minority	438/450	Yes	171	39.0	204	45.3
Schooling	441/450	Basic education or less	21	4.8	50	11.1
(mother/responsible adult)		Highschool or less	348	78.9	247	54.9
		At least one year of tertiary education	72	16.3	153	34.0
Use of pesticides	437/450	Yes	12	2.7	214	47.6
Smoking	437/450	Yes	3	0.7	113	25.1
(pregnant/mother of the child)		No	239	54.3	337	74.9
		Quit smoking (6 months to 1 year ago)	198	45		
Comorbidity	333/450	Yes	54	16.2	55	12.2
Living on a paved street	443/449	No	86	19.4	77	17.2
Drinking water	443/450	Tap water	173	39.0	64	14.2
		Rural drinking water or well water	11	2.5	4	0.9
		Bottled water	259	59.5	382	84.9
Fish consumption (last 3 days)	443/450	Yes	136	30.7	72	16
Living in an exposed area/beneficiary law	443/410	Yes	43	9.7	72	16
Change of address since birth	450	No			156	34.7

**Table 2:** Concentration of inorganic arsenic in urine of pregnant women (2013–2016) and children (2023) in Arica.

	Measure 1 (Pregnant Women)		Measure 2 (Children)	
	Inorganic Arsenic (*)	Inorganic Arsenic (*) Corrected by Creatinine	Inorganic Arsenic (***)	Inorganic Arsenic (***) Corrected by Creatinine
	(*n* = 443)	(*n* = 405)	(*n* = 450)	(*n* = 408)
	µg/L	µg/g **	µg/L	µg/g **
P25	10	13.1	9.0	11.7
P50	15	17.0	14.6	16.3
P75	23	23.0	20.8	23.0
P90	33	29.8	29.3	31.8
P95	41	38.9	35.9	36.5
Media	18.6	18.9	17.1	19.0
Standard deviation	14.8	9.4	14.8	11.9
Minimum	2.5	3.4	2.5	4.0
Maximum	126	72.8	202.0	146.4
Geometric mean	14.2	16.9	13.5	16.6
Pregnant woman living in an exposure area (*n* = 33)		17.0		18.5
Children with a parent who is a beneficiary of the Polymetallic Law (*n* = 55)		17.0		15.4

* inorganic arsenic and its methylated metabolites; ** inorganic arsenic adjusted for creatinine when creatinine is >0.3 and <3.0 gr/L; *** sum of inorganic arsenic (AsIII + AsV) + MMA + DMA.

**Table 3:** Bivariate analysis of creatinine-adjusted inorganic arsenic concentration (µg/g) in relation to sociodemographic, health, and arsenic exposure factors in pregnant women (2013–2016) and their children (2023).

		Pregnant Women				Children			
		*n* (%)	Median	IQR	*p* Value	*n* (%)	Median	IQR	*p* Value
Age of the mother	Under 20 years of age	55 (13.6)	17.2	12.3–23.2	0.884				
	20 to 39 years old	341 (84.2)	17.0	13.2–22.7					
	40 years old or older	9 (2.2)	16.7	15.7–23.9					
Age of the children	7 years old					127 (31.1)	17.3	13.3–23.8	0.026 *
	8 years old					216 (52.9)	16.8	11.1–24.1	
	9 years old or older					65 (15.9)	14.7	11.5–19.3	
Sex	Female					210 (51.5)	16.7	11.7–22.8	0.985
	Male					198 (48.5)	16.0	11.7–23.8	
Body mass index	Malnourished				0.926	19 (4.7)	13.5	10.8–21.4	0.679
	Normal weight	59 (14.6)	17.1	13.1–23.1		155 (38.2)	16.3	11.4–23.8	
	Overweight	153 (38.0)	17.2	13.2–22.6		96 (23.7)	17.4	11.6–24.0	
	Obese	191 (47.4)	16.5	12.8–23.1		136 (33.5)	16.1	12.4–22.7	
Belongs to ethnic	Yes	154 (38.5)	18.4	13.6–25.4	0.017 *	185 (45.3)	17.8	13.0–24.2	0.009 *
Schooling (mother/	Basic education or less	19 (4.7)	19.4	13.5–24.1	0.220	43 (10.5)	21.1	10.8–28.2	0.029 *
responsible adult)	Highschool or less	315 (78.2)	17.1	13.0–23.5		222 (54.4)	16.8	12.3–23.8	
	At least one year of tertiary education	69 (17.1)	15.5	13.1–20.4		143 (35.1)	15.0	10.8–21.4	
Use of pesticides	Yes	9 (2.3)	21.2	13.1–22.6	0.295	194 (47.6)	18.8	12.0–24.9	0.006 *
Smoking (pregnant/mother of the children)	Yes	3 (0.8)	25.8	16.7–28.6	0.112	100 (24.5)	15.1	11.2–22.7	
	No	212 (52.7)	17.9	13.3–23.1		308 (75.5)	16.7	11.8–23.2	
	Quit smoking (6 months to 1 year ago)	187 (46.5)	15.8	12.4–22.6					
Comorbidity	Yes	49 (16.3)	16.4	13.9–22.6	0.925	51 (12.5)	14.3	11.8–22.1	0.608
Living on a paved street	No	78 (19.3)	17.9	13.7–23.5	0.442	69 (17.0)	19.1	13.3–28.2	0.025 *
Drinking water	Tap water	158 (39.0)	17.6	13.6–23.5	0.014 *	57 (14)	17.4	13.3–24.7	0.005 *
	Rural drinking water or well water	9 (2.2)	21.2	20.2–35.3		4 (1.0)	33.7	30.0–64.2	
	Bottled water	238 (58.8)	16.4	12.5–22.6		347 (85.1)	16.0	11.5–22.2	
Fish consumption (last 3 days)	Yes	136 (30.8)	17.3	13.6–23.6	0.231	61 (15.0)	17.4	12.9–23.7	0.468
Living in an exposed area/beneficiary law	Yes	39 (9.7)	17.7	13.6–24.2	0.536	61 (14.9)	16.3	11.7–24.8	0.868
Change of address since birth	No					266 (65.2)	17.4	11.7–23.9	0.075

IQR = interquartile range, * *p* < 0.05.

**Table 4:** Linear mixed model for inorganic arsenic in urine measurements 1 and 2 (log-transformed adjusted for creatinine).

		Estimates (%)		
Variables		(exp(β) − 1) × 100	*p* Value	Confidence Interval (95%)
Drinking water	Tap water	Ref		
	Rural drinking water or well water	35.18	0.084	−4.00 to 90.34
	Bottled water	−8.25	0.037 *	−15.36 to −0.54
Living on a paved street	No	−4.98	0.303	−13.78 to 4.72
Belongs to ethnic	Yes	8.64	0.037 *	0.49 to 17.45
Schooling (mother/ responsible adult)	Basic education or less	Ref		
	Highschool or less	−4.02	0.553	−8.61 to 1.02
	At least one year of tertiary education	−13.67	0.042 *	−25.06 to −0.56
Use of pesticides	Yes	6.04	0.150	−2.09 to 14.84
Change of address since birth	No	2.07	0.614	−5.76 to 10.55
Age of the children		−2.30	0.416	−7.64 to 3.34

* *p* < 0.05.

## Data Availability

Data are not publicly available due to ethical restrictions.

## Supplementary Materials

The following supporting information can be downloaded at: https://www.mdpi.com/article/10.3390/toxics13030215/s1, Figure S1: Comparison of the distribution of inorganic arsenic concentration from measurement 1 in pregnant women in the 2013–2016 baseline sample (*n* = 1644) and the subsample of mothers who participated with their child in the 2023 subsample (*n* = 443); Table S1: Comparison of sociodemographic characteristics and inorganic arsenic concentration (μg/L) from measurement 1 in the baseline sample of pregnant women 2013–2016 (1644) and the subsample of mothers who participated with their child in subsample 2023 (*n* = 443).
