# Supplementary material for: Arsenic Exposure During Pregnancy and Childhood: Factors Explaining Changes over a Decade
Source: Toxics. 2025 Mar 16;13(3):215. doi: 10.3390/toxics13030215 (PMC11945348; doi:10.3390/toxics13030215)
Supplement: Supplementary file 1 [file toxics-13-00215-s001.zip › toxics-3441860-supplementary.pdf]

Suplementary Material

Table S1: Comparison of sociodemographic characteristics and inorganic arsenic concentration (µg/L) from measurement 1 in the baseline sample of pregnant women 2013-2016 (1,644) and the subsample of mothers who participated with their child in subsample 2023 (n=443).

		Base study n(%)	Sample n(%)
Number of individuals		1,644	443
Inorganic Arsenic	Median (IQR)	15 (9 a 23)	15 (10 a 23)
	Lower than 35 µg/L	1510 (91.9%)	407 (91.9%)
	35 µg/L or higher	134 (8.2%)	36 (8.1%)
Year of enrolment	2013	92 (5.6%)	35 (7.9%)
	2014	617 (37.5%)	144 (32.5%)
	2015	930 (56.6%)	262 (59.1%)
	2016	5 (0.3%)	2 (0.5%)
Belongs of a ethnic	Yes	598 (36.8%)	171 (39.0%)
Schooling	Basic education or less (<8)	35 (2.2%)	9 (2.0%)
	High School or less (8-12)	1337 (82.3%)	360 (81.6%)
	At least one year of tertiary education (13+)	253 (15.6%)	72 (16.3%)
Age	Median (IQR)	26 (21-31)	26 (21-31)

IQR=Interquartile range

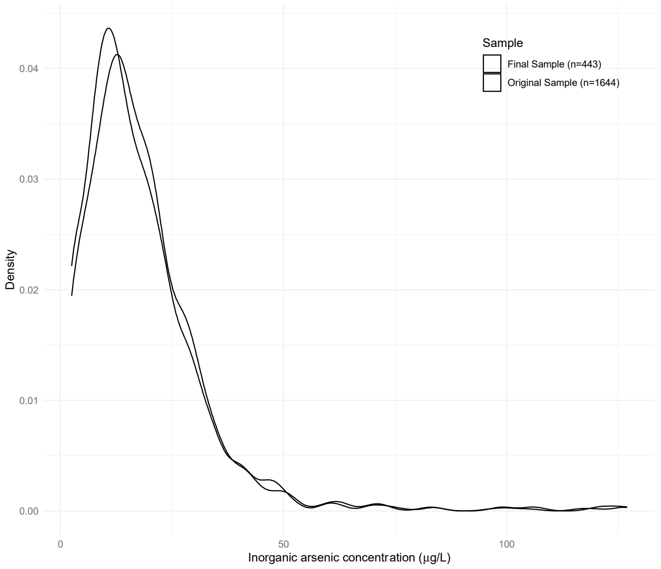

Figure S1: Comparison of the distribution of inorganic arsenic concentration from measurement 1 in pregnant women in the 2013-2016 baseline sample (n=1,644) and the subsample of mothers who participated with their child in the 2023 subsample (n=443).
